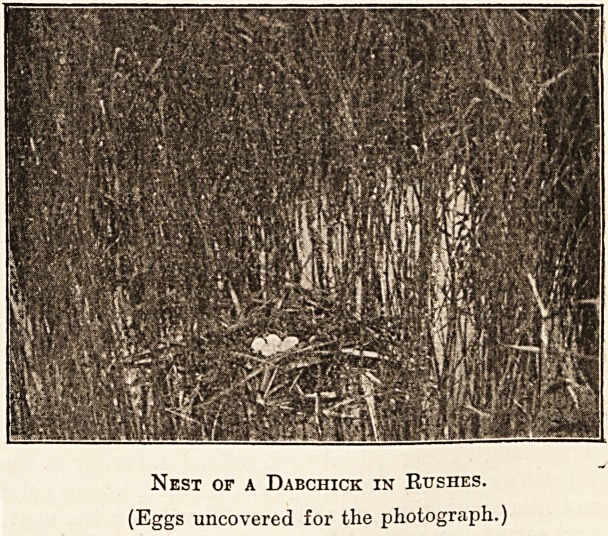# A Study of Dabchicks

**Published:** 1909-07-03

**Authors:** 


### The Practitioner's Relaxations.

### A STUDY OF DABCHICKS.

Among fishermen the Dabchick—or, to give him his full title, the Little Grebe—is a much execrated bird. His particular villainy is the consumption of the spawn and the young fry of the fish which the angler desires to reserve for his own special delectation. I would deny the charge if I could, for I like the little scoundrel, but unfortunately my own experience proves its accuracy. In the stomach of a dabchick shot during the spawning season I have found evidence enough to hang the whole tribe, and I have also seen a dabchick who had choked himself in his endeavour to swallow a stickleback. There is thus no doubt about his sins, and he is guilty of another minor offence most exasperating to the dry-fly fisher—I speak feelingly as one who has suffered—which is his frequent mimicry of a rising fish.

Male Dabchick Sitting.

Imagine a day when rising fish are scarce. You are standing on the alert for those spreading concentric ripples which betoken a moving trout, when some subconscious sense bids you turn, and, behold—the very thing you were looking for. "A good fish, too," you mutter, with a hasty glance to see that the fly is all right. Then the stalk begins, and perhaps you are almost within throwing distance when an inquiring little head pops up from the depths, and the fraud is revealed. This is all very aggravating, especially on a bad day; but still, the dabchick is only doing in another fashion what you yourself are doing—trying to catch fish. And so we come back to his first offence, which is aggravated by the success with which he fishes in all weathers.

Personally I have no serious quarrel with the dabchick. I admit that one may have too many of him, but my experience, in fishing on water wherein the dabchicks are not destroyed as vermin, is that trout abound in spite of him; whence I conclude that there are enough fish in the water for both of us. Moreover, I find him, in common with the many other birds that frequent the banks of our chalk streams, a perpetual source of amusement when fish are not rising.

To gain anything like an intimate acquaintance with the dabchick in his natural haunts—I except such places as the ornamental water in St. James's Park, where he is so tame as to be almost domesticated—it is necessary to conceal oneself thoroughly, near the nest. There is no difficulty in finding the nest when once one knows what to look for. It consists simply of a platform of river weed floating on the water, either on top of a mass of growing weed which keeps it in position, or cunningly moored to growing rushes or the overhanging branches of a tree—usually a willow or alder. The platform generally takes some time to build, or perhaps it would be more correct to say that it is frequently built some time before it is needed. In due course some four to six white eggs are laid upon it, and these are always carefully covered over with loose weed and other river litter, when the bird leaves the nest. This process serves a double purpose. The covering conceals the eggs, whose whiteness would inveitably attract attention if they were exposed; and the fermentation of the rapidly decaying weeds produces heat sufficient to keep the eggs warm for a long while. Another effect of this perpetual contact with decaying vegetable matter is a remarkable discolouration of the eggs, which by the end of the period of incubation are generally of a pale chocolate or café-au-lait colour.

Although the birds can safely leave their eggs, when well covered, for a considerable period without the risk of their becoming chilled, they do not willingly do so. Both birds sit, the female being the more assiduous of the two, and the eggs are only deserted on the suspicion of approaching danger. The birds are, however, very quick to take alarm, and are endowed with a very keen sense of hearing, whereby they can detect the human voice or human footfall at a considerable distance, and leave the

Nest of a Dabchick in Rushes.

(Eggs uncovered for the photograph.)

nest as soon as assured that the alarm is not a false one. For this reason it is extremely difficult to stalk the bird on the nest, and it is almost impossible to force her to leave the eggs uncovered. Given plenty of time she will cover them very thoroughly with a convex pile of weed, but if she is taken unawares she will, with three or four extremely rapid thrusts of her bill, throw enough weed over them to conceal them from view, and will then dive as silently as a vole into the water, reappearing only when she is some twenty or thirty yards at least from the nest.

I have said that in the act of incubation the female is the more assiduous of the two. In so saying, I must admit that I am stating as a fact what is, strictly speaking, only my own opinion. In the course of a prolonged study of a pair of these birds at their nest I soon learned to distinguish the birds apart. One of them sat very persistently: the other which I took to be the male, did a short spell of duty now and again. The chief difference which distinguished the male, as I shall call him, from the female was his slightly stouter body and longer head and bill. Although he was rather slack in the matter of incubation, and very ready to leave the nest on the slightest pretext of alarm, he was prettily attentive in other ways. From time to time he would visit the female as she sat, announcing his approach with a soft tremulous call; would swim round the nest repeating this call very gently with an occasional answer from his mate; and at intervals would dive around his home, returning each time with a wisp of fresh weed in his bill, which he piled carefully around the sides of the nest, and so added daily to its height and bulk. It is the practice of these birds to cover the eggs more heavily as incubation proceeds, so that towards the end of the period the nest when covered may attain at the centre a height of some six to nine inches from the water level.

It was quite obvious to me that a clear understanding existed between the two birds on the subject of what I will call for convenience "changing guard." What I mean by this is that when the time came there was never any degree of doubt in the mind of either bird. The sitting bird would leave the nest with the eggs exposed, immediately upon the appearance of "the relief," and the mate would promptly take up the post of duty; but how the matter was settled I could not discover. During the majority of the visits of the male, the female remained steady at her post, answering his call from time to time, or, with extended neck, helping to arrange the fresh weed he brought up from the deep water. Then, on another occasion, at the first sound of his voice, she would walk to the edge of the nest and dive out of sight, usually without so much as a sound of recognition. The male, I observed, always retired in favour of the female as soon as she reappeared. He evidently did not like incubation, was always fidgetty on the nest, and seized any pretext for retiring from it. I always suspected that his extreme timiditty about sounds in the neighbourhood was for the most part simulated.

In approaching and in leaving the nest these birds were models of caution. They approached always from beneath, appearing suddenly under the bank close by, or, on occasions, bobbing up actually through the outer fringe of the nest itself. Similarly in leaving the nest they invariably dived, either from the nest or at some spot within a few feet of it, and only came to the surface when they had reached the cover of (as a rule) the opposite bank. They are extremely powerful divers, as, indeed, they must be to live (for their food is taken almost entirely under water), and if surprised in a narrow open stream, with little available cover, it is possible, by following him along the bank, to make him swim really extraordinary distances under water.

It is said of the dabchick, as of most regularly aquatic birds, that the old birds carry their young on their backs. I can well believe that this is true, though I have never actually seen it; but I doubt whether it means much more than that the young now and then climb on to the backs of their parents, much as young chickens often sit on the back of the hen in a farmyard. I do not think it likely that the young are at all dependent on such assistance. At a very tender age they are expert swimmers and divers, and I think that, like young ducks and moorhens, they can probably swim as soon as they are hatched, but I have never succeeded in seeing the young actually leave the nest. The particular nest which was the object of my most prolonged investigations was unfortunately destroyed when the eggs must have been within a day or two of hatching out.

I have frequently, when fishing, watched young dabchicks, obviously not more than two or three days old, swimming and diving about in a most masterly fashion, and have been astonished at their hardihood and the impunity with which they dive for concealment under the densest patches of weed, always managing to find an opening through which they can thrust their bills and breathe in security, until a movement of the suspected enemy on the bank warns them to dive again and take cover elsewhere.

I will leave it to the scientific zoologist to discuss the anatomical peculiarities of the dabchick's leg. The method of connection between the fibula and the tibia is, I believe, unique; but I am more interested in the habits of the bird than in its construction. I have admitted his vices, but I venture to plead with the most ardent sportsman to deal gently with him. Seen at a distance he is a dull looking bird enough, and the frequent excursions under the water, which have earned him his popular name, while engaging enough in themselves, are to the fisherman painfully suggestive of the depredations which have made him notorious. But I venture to think that many of his traducers would be inclined to relent if they knew him more intimately. This is not really a matter of great difficulty. All that is necessary is to locate a nest, rig up a rough shelter of three hurdles littered over with brushwood or grass, leave it alone till the birds have become used to it, and then, on a blank day, conceal oneself in it and watch them at leisure. It will be found that the dabchick is not lacking in good looks; while the study of his home life and domestic habits proves so absorbing that his iniquities will, for a time at least, be forgotten and perhaps forgiven.

## Figures and Tables

**Figure f1:**
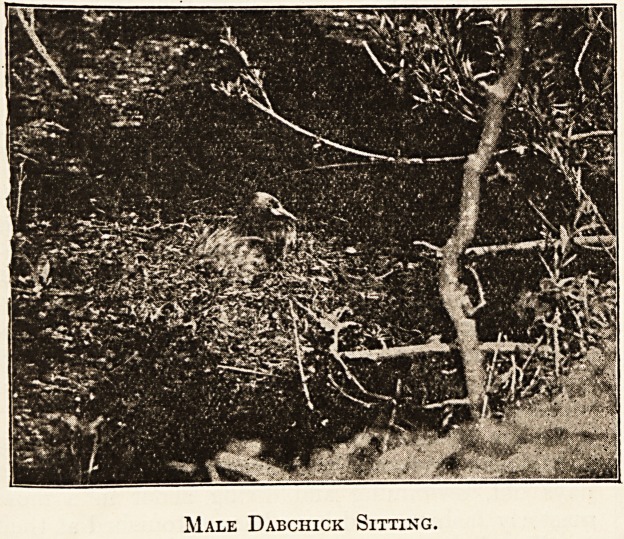


**Figure f2:**